# A new perspective of frozen shoulder pathology; the interplay between the brain and the immune system

**DOI:** 10.3389/fphys.2024.1248612

**Published:** 2024-03-29

**Authors:** Santiago Navarro-Ledesma, Dina Hamed-Hamed, Leo Pruimboom

**Affiliations:** ^1^ Department of Physical Therapy, Faculty of Health Sciences, Campus of Melilla, University of Granada, Melilla, Spain; ^2^ University Chair in Clinical Psychoneuroimmunology, University of Granada and PNI Europe, Melilla, Spain; ^3^ Clinical Medicine and Public Health PhD Program, Faculty of Health Sciences, University of Granada, Granada, Spain

**Keywords:** frozen shoulder (adhesive capsulitis), shoulder pain, shoulder condition, autoimmune disorder, endocrinological disease, low grade inflammation, psychosocial factors

## Abstract

Frozen shoulder (FS), also known as adhesive capsulitis of the shoulder (FS), is a fibrotic inflammatory process of unknown etiology whose main symptoms are pain, stiffness and the loss of joint mobility. These symptoms may be associated with pathologies such as diabetes, Dupuytren’s syndrome and the prevalence of today’s sedentary lifestyle. This literature review provides an overview of the epidemiology and pathogenesis of this pathology, as well as the mechanisms of lowgrade chronic inflammation and infection, insulin resistance, and omics-science associated with it. We also propose a new hypothesis related to the possibility that the GABAergic system could play a decisive role in the development of frozen shoulder and that therefore diabetes type 1, endocrinological autoimmune disorders and frozen shoulder are connected by the same pathophysiological mechanisms. If that is true, the combined presence of psycho-emotional stress factors and pathogenic immune challenges could be the main causes of frozen shoulder syndrome. Finally, we propose a series of possible intervention strategies based on a multifactorial etiological and mechanistic concept.

## 1 Introduction

Frozen shoulder (FS) or adhesive capsulitis of the shoulder (FS) is a disorder characterized by its inflammatory and fibrotic process (Pietrzak, 2016). Codman first included the term “frozen shoulder” in 1934 (Dias et al., 2005). The predominant symptoms of this pathology in people suffering from it are stiffness, pain and a limitation in the range of active and passive mobility of the glenohumeral joint (Hannafin and Chiaia, 2000; Hagiwara et al., 2018). These symptoms have impact on personal and professional life and disturb people from performing their normal daily activities, affecting their quality of life (Chen et al., 2017). The disability in the shoulder can last over a period of one or more years during which range of motion is slowly restored in 60% of the affected population (Sarasua et al., 2021).

The other 40% of patients develop a chronic disorder (Buchbinder et al., 2007) and become increasingly more limited in their daily functions (Walmsley et al., 2014a; Chen et al., 2017). Interesting is that the quality of life seems to be solely related with purely the loss of motion of the affected shoulder and as such a successful treatment would improve both physical performance and quality of life (Fernandes, 2015).

It is of outmost importance to understand the reasons why FS resolves in 60% and persist in 40% of affected patients. Several factors seem to operate simultaneously in patients in which FS does not resolve and are of psychological, functional, etiological, and biological character. A retrospective study concerning the success rate of arthroscopic capsular release and relationship with functional and psychological factors showed that traumatic etiology, depression and anxiety decreased success rate after operation (Galasso et al., 2023). Another study with 210 patients (F/M = 130/80), investigated the outcome of consecutive physiotherapy, cortisone injection and, when necessary, arthroscopic release, in patients with FS and the impact of diabetes and obesity on that outcome (Barbosa et al., 2019). Interestingly, the study showed no association of BMI and successful outcome of FS treatments whereas patient with diabetes showed worse results when treated with physiotherapy and cortisone injection. The authors of this study refer to diabetes as a known risk factor for FS and conclude that mechanistic prove still lacks to explain the significant impact of diabetes on onset and resolve of FS (Barbosa et al., 2019). They refer to the fact that glycosylated Hb does not seem related with FS and so glycation and the forming of advanced glycolated end products cannot explain mechanistically the relationship between FS and persistent FS. Other explanations are therefore needed. Later we will describe a new hypothesis about the way FS develops and persists.

The common opinion about the pathophysiology of FS is based on a “inflammation-fibrotic cascade’ theory, characterized by fibroproliferative tissue fibrosis, whereby fibroblasts, producing predominantly type I and type III collagen and transform into myofibroblasts (a smooth muscle phenotype). This process is further accompanied by inflammation, neoangiogenesis and neoinnervation (Millar et al., 2022). Neoinnervation and the change of tissue morphology could explain the main hallmarks of FS, meaning pain and loss of range of motion (Akbar et al., 2019a). Epidemiological data reflect a prevalence of FS of 3%–5% in the general population (Sarasua et al., 2021).

The syndrome consists of three phases (Walmsley et al., 2014a; Cho et al., 2018): i) the painful freezing phase in which pain precedes a loss of range of motion lasting 10–36 weeks (Walmsley et al., 2014b; Zhang et al., 2021); ii) the state of freezing or adhesive phase, lasting 4–12 months where pain gradually reduces while the range of motion stays impaired (Walmsley et al., 2014b; Zhang et al., 2021); and iii) the thawing or regression phase where range of motion improves progressively (Walmsley et al., 2014b; Zhang et al., 2021). However, 40% of patients do not recover normal range of motion and keep experiencing symptoms and that makes this classification still contentious (Millar et al., 2022).

The course of FS and its impact on physical wellbeing, mental health, together with a sustained loss of productivity and global economy has been shown (Bouaicha et al., 2020). Although a common opinion about the pathophysiology exists (the above-mentioned cascade), the disparity of results observed in the literature regarding the etiology of FS, suggests that there remain mechanistic lagunes in the pathophysiology and etiology of FS (Austin et al., 2014).

New insights about the possible etiology of FS come from a recent study (de Mello et al., 2023) that shows a significant increase of FS cases during the COVID-19 pandemic. They investigated retrospectively 1.983 patients that suffered from FS and compared the number of patients that became affected from March 2019 to February 2020 and from March 2020 to February 2021 (de Mello et al., 2023). During the pandemic the incidence of FS was 2.41-fold higher compared to the previous year (de Mello et al., 2023). Data that confirmed the results of other epidemiological studies were related with a 4-fold increase of FS in patients suffering from diabetes (Whelton and Peach, 2018). The same holds for the possible association between hypothyroidism and the incidence of FS which in this study shows a 5-fold increase, again in coherence with earlier published data (Cohen et al., 2020).

The new findings are mainly related with psycho-emotional factors. The individuals that suffered from depression and anxiety had, respectively, 8.8 (*p* < 0.001) and 14 (*p* < 0.001) times and significant greater risk on the development of FS (Whelton and Peach, 2018). The results of an earlier systematic review (Brindisino et al., 2022) already emphasized on the impact of depression and anxiety on physical dimensions such as shoulder pain, range of motion and pain perception. These data suggest that ‘toxic’ emotions and ‘toxic’ thoughts (Pruimboom, 2023) could be part of the multifactorial etiology of FS and further research should elicit possible mechanisms of how exactly these ‘yellow flag’ factors could cause FS.

Genetic susceptibility as risk factor for FS is also proposed and suggested by results of a genome-wide association study by Scott et al. (Scott-solomon et al., 2022). Three polymorphisms of genetic loci related with cell proliferation and production of extracellular matrix and collagen fibers seem to be as associated with FS, diabetes and hypothyroidism and so genetic susceptibility should be included in the list of possible risk factors (Scott-solomon et al., 2022). One of the loci as a risk factor for FS is the Wnt gene (Scott-solomon et al., 2022). A polymorphism of the Wnt gene has also been found in people suffering from morbus Dupuytren, giving a possible explanation for the relationship between morbus Dupuytren and FS (Dolmans et al., 2011).

Further confusion about the definite causes of FS and the mechanisms behind it is caused by the fact that there exists a great disparity in success rates by interventions based on the known epidemiologic, etiological and mechanistic data known thus far (Cohen et al., 2016; Cho et al., 2019; Mertens et al., 2022). These disparities make it legitimate to state that the best way to treat people with FS is primary prevention.

Psychological factors were already identified as risk factors for the development of FS and for worse outcome in patients who underwent chirurgical repair of a rotator cuff injury (Aïm et al., 2022). The authors of the latter study conclude that although significant worse outcome was measured for patients suffering from anxiety and depression, it was not clear if the psychological state was cause or consequence (Aïm et al., 2022). Anxiety, depression and pain, the latter a hallmark of FS, do not only share psychological characteristics, but also biological ones.

Spontaneous pain, as initiating symptom for idiopathic FS, is often part of a cluster of syndromes including depression, pain and chronic fatigue (Mokhtari et al., 2019). Several pathways have been described to explain the comorbidity between the three syndromes, although the common opinion about the connections is mostly based on the presence of a chronic neuroinflammation causing hippocampal atrophy, accompanied with decreased neurogenesis, decreased neuroplasticity and less presence of neurotrophic factors (Raison et al., 2006; Jain et al., 2011; Mokhtari et al., 2019). Causes of neuroinflammation range from endotoxemia (De and Pruimboom, 2015; Mohammad and Thiemermann, 2021), and metabolic syndrome (Van Dyken and Lacoste, 2018) via acute brain trauma and stroke (Leung et al., 2007) to sleep disturbances (Zhu et al., 2012a) and high calorie diet (Robbins and Solito, 2022). Stroke and acute brain trauma have also been associated with FS (Leung et al., 2007) and the same holds for sleep disturbances (Jankowska-Polańska et al., 2021). Overall, psychological and immunological factors are related with onset and prognosis of FS and so treatment should account interventions improving functioning of the brain, the immune system and the shoulder.

If we could identify the sum of non-confounding risk factors as cause of FS than the use of for instance lifestyle medicine would be indicative. Primary prevention is more than early detection of a disease. Nevertheless, measurements that help to recognize the disease in its early stage before symptoms appear and fibrosis sets in, helps to slow the development of FS and supports faster healing (Pietrzak, 2016). In this regard, metabolic factors (diabetes mellitus and thyroid disorders, metabolic syndrome), autonomic symptoms and pain sensitivity may contribute to the prognosis in terms of shoulder pain, disability, and quality of life in patients with FS (Nathan, 2002). Therefore, inflammation control has been proposed to be the best option when the etiology of a pathology is unknown (Hand et al., 2008). The diagnosis of frozen shoulder is classified into primary and secondary. Primary FS is characterized by a progressive and painful loss of the active and passive mobility range in the shoulder with its causes being unknown (Walmsley et al., 2014b); secondary FS is of a known intrinsic or extrinsic cause with stiffness in the shoulder being established for instance after surgery (Hannafin and Chiaia, 2000).

The care of musculoskeletal health is often based on the recognition of patient-reported findings, physical examination and evidence of an initial inflammatory state leading to fibrosis (Hand et al., 2008).

However, some patients with FS (between 7% and 50%) have symptoms for a long period of time with 6% having them for more than 7 years (Eljabu et al., 2016), which again can be explained by an incomplete understanding of frozen shoulder’s etiology (Mertens et al., 2022). In this sense, FS is currently understood in a multifactorial way, in which chronic low-grade inflammation (LGI), alterations in glucose metabolism (especially insulin resistance with compensatory hyperinsulinemia), chronic ischemia and endotoxemia are important factors of great scientific interest. Perhaps adding omics sciences could shed light on the understanding of the mechanisms and causes that lead to FS and we will therefore add some data related with metabolomics and proteomics (Nathan, 2002; de la Serna et al., 2021). We will further propose a new perspective of the etiology and possible treatment of patients suffering from FS through the description of the interplay of the brain and immune system in people suffering from FS, diabetes type 1 and even morbus Hashimoto.

## 2 Epidemiology

The prevalence of FS in the entire population is 2%–5% (Dias et al., 2005; Hand et al., 2008; Austin et al., 2014). Most patients suffering from FS are women (70%) (Hannafin and Chiaia, 2000) with the most frequent age range being between 40 and 60 (Hand et al., 2008). Although the exact etiology is unknown, the risk factors are: diabetes, Dupuytren’s syndrome, heart and neck surgery, Parkinson’s disease, smoking, thyroid disease and chronic regional pain syndrome (Bridgman, 1972; Hannafin and Chiaia, 2000; Thomas et al., 2007; Chan et al., 2017).

FS has been clearly demonstrated to be associated with diabetes (Thomas et al., 2007; Austin et al., 2014; Chan et al., 2017), with type 1 diabetes being the most frequent risk factor for its development. Its incidence in the diabetic population is 10%–36% higher than in the general population, which indicates that poor glucose control is related to the development of FS in the long term (Thomas et al., 2007). A key factor in this association is the level of hemoglobin (Bridgman, 1972). In the literature, there is a hypothesis which suggests that hyperglycemic levels associated with diabetes can incite changes in the collagen of the joints thereby unleashing fibrotic and inflammatory variations, and these changes in the joint have been observed in pathological studies of the disease (Austin et al., 2014). Chronic hyperglycemic levels increase the possibility of the production of advanced glycation end products which are known to cause cross linking of collagen fibers that can result in fibrosis of the capsules of the shoulder (Li et al., 2015). One of the most susceptible substances for glycation caused by chronic hyperglycemia is hemoglobin (Malka et al., 2016) and therefore HbAc1 could be a valid parameter of AGE products as part of the etiology of FS. A relevant study including 201,531 patients with diabetes of which 1,150 suffered from frozen shoulder did not support any association of HbAc1 with the incidence of FS (Yian et al., 2012). This study confirms that people suffering from diabetes have an increased risk for FS but glycation of HbAc1 does not seem to have influence on that risk (Yian et al., 2012). It is surprising that people suffering from diabetes type 1 are at greater risk for suffering FS than patients suffering from diabetes type 2 (Thomas et al., 2007). Both groups of patients show repetitive hyperglycemia periods and both show signs from inflammation and metabolic disturbances at the level of lipid physiology including LDL, VLDL and triglycerides values in the bloodstream (Razi et al., 2017; Tell et al., 2020). Dyslipidaemia is part of the pathophysiological changes in FS and therefore the metabolic state resulting from diabetes one and two could explain partly the association between diabetes and FS. What it does not explain is the difference between the impact of diabetes type 1 and 2 on FS. COVID-19 has perhaps given a new perspective to explain this difference. FS is also associated with axial spondylarthritis (axSA), according to a study investigating 2,859 patients suffering from axSA (Huang et al., 2020). SA is associated with increased risk and early onset in patients human leukocyte antigen B27 (HLA-B27)-positive (Huang et al., 2020). A recent meta-analysis and systematic review found that FS is also strongly associated with the presence of HLA-B27 (Prodromidis and Charalambous, 2016). These findings further support the possibility that FS has an auto-immune origin or shares certain pathophysiological mechanisms with all the auto-immune diseases with which FS risk is associated.

One of those mechanisms could be related with the GABA-ergic dependent nervous system. Diabetes type 1 is considered an autoimmune disease and the main autoimmune response is against glutamic acid decarboxylase (GAD65) (Towns and Pietropaolo, 2011). GAD65 is a super autoantigen with high level of presence in the brain (Nataf, 2017).

**FIGURE 1:**
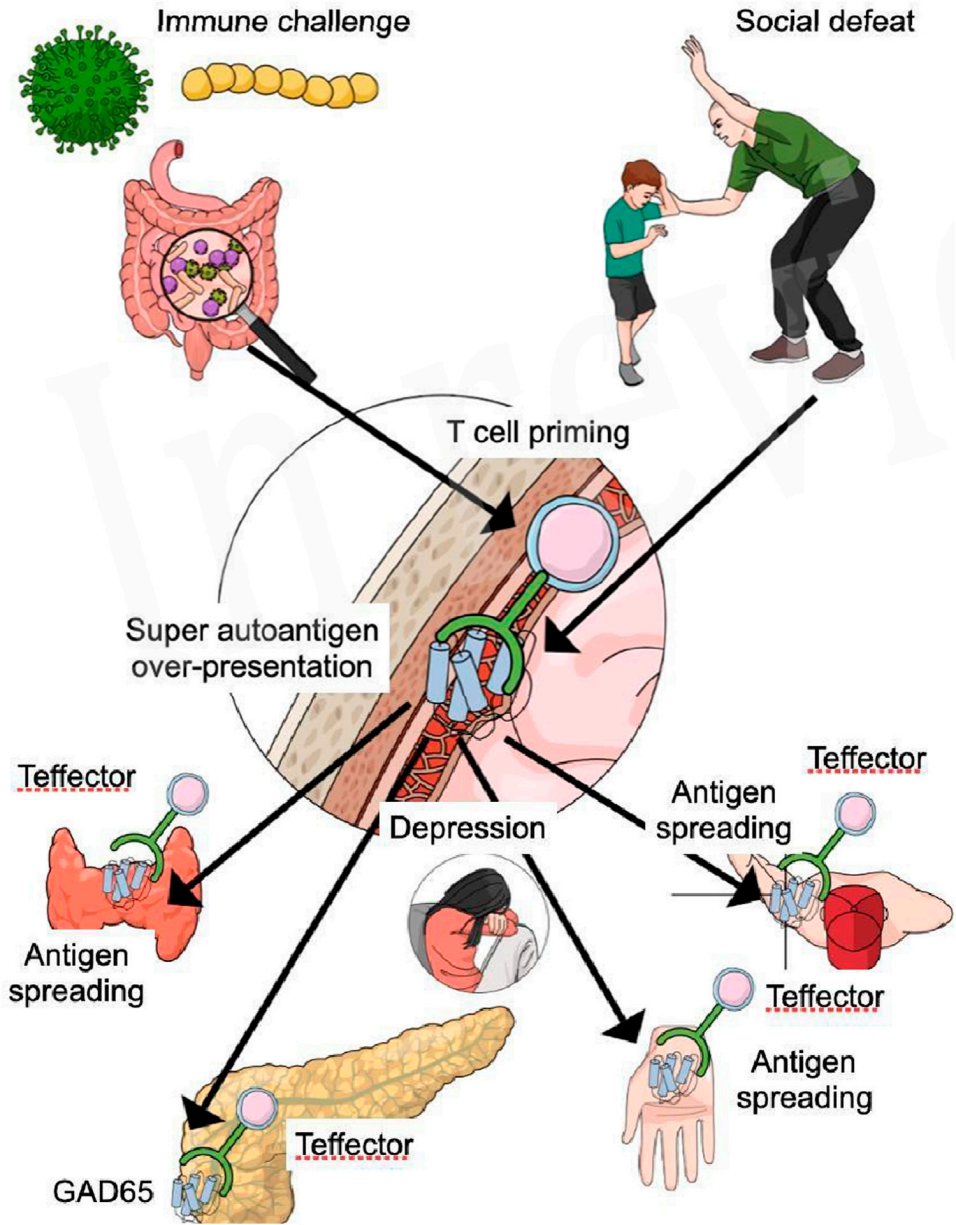
Social defeat and psycho-emotional distress lead to increased presentation of brain super autoantigens, which are presented to pro-inflammatory T lymphocytes present in the meninges of the brain. Immune challenges such as virus, bacteria and leaky gut, sedentary lifestyle, and obesity, produce a LGI and activity of both the innate and acquired immune system. The heightened activity of the T lymphocytes increases the susceptibility for the development of Teffector cells primed for multiple super autoantigens like GAD65. Active Teffector cells respond to GAD65 (antigen) presented by antigen presenting cells in the meninges inducing the primus movens of a possible autoimmune reaction. Teffector cells primed for GAD65, migrate to the peripheral body and specifically the pancreas, where they find a high level of GAD65 inducing a severe auto-immune response that ultimately leads to diabetes type 1. Antigen spreading to specific sites attract Teffector cells into the susceptible shoulder (non-dominant neglected), the thyroid gland, and the hand muscles, whereas immune activity in the brain causes a neuroinflammation, leading to depression, fear and anxiety.

GAD65 is a synaptic enzyme that catalyzes y-aminobutyric acid (GABA) from glutamate. Its expression in inhibitory terminals is essential for functioning of the GABAergic system (Mende et al., 2016). The immune reaction against GAD65 resulting in the production of antibodies is not only evidenced in diabetes type 1 but also in several central nervous system disorders grouped as GAD-antibody spectrum disorders (Tsiortou et al., 2021). These disorders are characterized by severe anxiety, depressed mood, and specifically anxiety and phobias for physical challenges (Tsiortou et al., 2021). The study of Pires (de Mello et al., 2023) showed that anxiety and depression during the COVID-19 pandemic increases the risk for FS with a factor 8.8 which is even greater than other associations (de Mello et al., 2023). GAD65 antibodies that can lead to diabetes type 1 and central nervous disorders are often a consequence of two synergistic risk factors, according to Nataf (Nataf, 2017). The simultaneous exposure of an immune challenge combined with psycho-emotional stress increases the risk of T cell priming against GAD65 and this can produce both diabetes type 1 and central nervous system disorders (Nataf, 2017). SARS-COV-2 has brought fear, anxiety an depression to many affected and non affected COVID-19 individuals (Kupcova et al., 2023), and these psycho-emotion state in combination with viral challenges could be responsible for an increases of antoantibodies GAD65 in persons already suffering from diabetes type 1. It is even possible that COVID-19 can elicit diabetes type 1 according to a case study by Genç (Genç et al., 2021). During the pandemic cases of hyperglycemia, diabetic ketoacidosis and diabetes type 1 increased and so SARS-CoV-2 seems to trigger or unmask DM1 (Wang et al., 2013). Could autoantibodies against GAD65, which are associated with diabetes type 1, brain disorders, anxiety, and depression, also have a significant role in the development of Frozen Shoulder (FS), especially considering its higher occurrence in patients with hypothyroidism and Hashimoto’s disease (Brindisino et al., 2022), and the elevated risk of endocrinological autoimmune diseases related to these autoantibodies (Gambelunghe et al., 2000)? The GABAergic system has multiple functions related with hallmarks of inflammatory diseases such as rheumatoid arthritis (Shan et al., 2023), pain (Jasmin et al., 2004), and GABA-disorders as seen in people suffering from autoimmune activity against GAD65 develop a stiff body disease (Tsiortou et al., 2021). These data, although speculative, support the development of the hypothesis that FS related with diabetes type 1 is of autoimmune origin. Antibody spreading is a known mechanism in autoimmune diseases and spreading of GAD65 has been evidenced in people suffering from diabetes type 1, although the level of auto-antibodies was not high enough to predict auto-reactivity in the infiltrated tissues (Söhnlein et al., 2000). Nevertheless, disturbances of the GABA-system could lead to fear of physical challenges and movement neglect is one of the evidenced risk factors for the development of FS (de la Serna et al., 2021) (Figure 1).

When eliminating the difference of incidence between DM1 and FS and DM2 and FS, it seems plausible that components of the metabolic syndrome and the metabolic syndrome as part of DM1 and two are (co) responsible for increased pro-inflammatory cytokine production (Austin et al., 2014), causing a state of chronic low-grade inflammation as part of the pathophysiology of FS (Hutcheson and Rocic, 2012). This chronic low-grade inflammation is further related with increased oxidative stress, caused by the production of excessive reactive oxygen species (ROS) and oxidative stress is also part of FS (Vicente, 2016).

### 2.1 A short resume of the literature related with the shoulder pathogenesis in FS

Stiffness, pain, and limited range of motion are common symptoms in people suffering from frozen shoulder (FS). The accumulation of advanced glycation end products (AGEs) in the shoulder, associated with insulin resistance, compensatory hyperinsulinemia, chronic hypoxia, and endotoxemia, are also characteristic (Bridgman, 1972). Cytokines play a fundamental role in the development of the disease and could possibly be used as diagnostic markers. Interleukin cytokine IL-1 is involved in key functions such as immune cell recruitment, cell proliferation and tissue destruction (Chen et al., 2017). The presence of activated mast cells, T and B lymphocytes and inflammatory mediators (including cytokines) found in synovial biopsies in patients suffering from FS, support the pathophysiological role of pro-inflammatory cytokines in FS (Bridgman, 1972). These cytokines further can cause persistent capsular fibrosis and thus, the development of FS. Next to infiltrated immune cells, fibroblasts and myofibroblasts are present in great amounts (Bunker et al., 2000), whose main function are the maintenance of the extracellular connective tissue matrix. Overactivation of myofibroblasts gives raise to formation of fibrotic tissue and tissue contraction, whereas they normally have an important repair function (Ramos et al., 2004). When the matrix contracts, pain and stiffness occur. Myofibroblasts and immune cells influence each other and by their cytokines and growth factors (Bunker et al., 2000).

## 3 Low grade inflammation and subclinical infection

Modern life is characterized by high calorie intake, sedentary lifestyle and many other toxic factors brought by the development of culture (Ramos et al., 2004). Culture should not been seen as some reaction on evolution, it is just its logical result (Ramos et al., 2004). The factors that killed humans millions of times have been responsible for the search for solutions and humans have achieved many benefits by using their intelligence. Today people in developed countries hardly die of starvation, dehydration, heat or cold because of respectively agriculture, a sanitary system, air conditioning and central heating (Al-Amrani et al., 2021). Nature shaped survival strategies and intelligence and the end-result is culture. The need for physical activity is almost fully eliminated by motorizing transport, food is brought by a courier and so too much food and a chronic lack of physical activity are logical consequences of culture as a result of nature (Ramos et al., 2004; Pruimboom, 2023). Culture, as result of nature, developed based on the ultimate law of evolution, meaning save energy for survival and reproduction (Casanova et al., 2023).

Modern lifestyle is a direct cause of many detrimental health effects and that probably means we have had too much of culture (Pruimboom et al., 2015). Factors such as light pollution, air pollution, sleep disturbances, alcohol abuse, smoking, sitting time, high calorie diet, increase loneliness and social defeat factors such a poverty, all produce a chronic stress response, with multiple consequences (McEwen et al., 2015). Chronic stress leads to activation of the immune system. Lifestyle can also directly cause inflammation by the production of a lifestyle associated molecular pattern that consists of increased LDL and uric acid. Both are immunogenic compounds producing a pro-inflammatory state (Zindel and Kubes, 2020). Chronic stress was recently defined as chronic irritation (Navarro et al., 2023) and chronic irritation produces low-grade inflammation that, on its turn, increases permeability of multiple mucosal barriers including the gut (Fasano and Shea-Donohue, 2005; de Punder and Pruimboom, 2015; Fasano, 2020; Zindel and Kubes, 2020; Navarro et al., 2023). The translocation of bacterial debris and other toxins to the blood stream, and present in those mucosal organs, are co-responsible for the observed low-grade inflammation (Fasano and Shea-Donohue, 2005; Fasano, 2020). During any type of inflammation barrier break-down is observed where any form of injury can start with low-grade inflammation and may lead to systemic inflammation (Bunker et al., 2014). LGI activates the stress systems chronically and cortisol, noradrenalin and pro-inflammatory cytokines can cause (severe) damage to tight junctions of systemic barriers (de Punder and Pruimboom, 2015). The blood-brain barrier, the blood-retinal barrier, the blood-nerve barrier, the blood-lymph barrier and the blood-cerebrospinal fluid barrier increase their permeability or are damaged by LGI, possibly causing a systemic inflammation of all vital organs (Bridgman, 1972; Rönnbäck and Hansson, 2019). The systemic inflammation caused by LGI can easily explain the presence of an inflammatory state in the frozen shoulder of affected people (Hagiwara et al., 2018). Some cytokines, such as HMGB1, have been shown to play a role in FS pathology; they are thought to be needed for the onset and perpetuation of FS since their release during stress bolsters the inflammatory tissue response seen in patients (Akbar et al., 2019a; de la Serna et al., 2021; Millar et al., 2022).

LGI can be produced by many chronic stress factors of both sterile and non-sterile character. Non-sterile etiological factors for FS include possible infiltration of bacteria that normally inhabit human skin, such as Propionibacterium acne (P. acne), that recently has been identified in people suffering from FS (Bunker et al., 2014; Cucchi et al., 2017). A recent study showed the presence of multiple alarmins (IL-33, S100A9, S100A8 and HMGB1) in the joint capsule in FS, and these alarmins are associated with an infective response against P. acne (Cucchi et al., 2017; Akbar et al., 2019b). P. acne could not only be a possible cause of FS, it is also the most frequent bacteria that causes infection in the affected shoulder after surgical procedures (Cher et al., 2018). Corynebacterium propinquum and *Streptococcus* epidermis are commonly found in the affected shoulder and are also part of etiological factors of disc herniation as shown in several studies (Lavergne et al., 2017). The postulated mechanism by which these bacteria reach the shoulder capsule is through the mouth, mainly during tooth brushing (Bhanji et al., 2002; Capoor et al., 2017). Oral dysbiosis, which mediates local and peripheral inflammatory pathologies, can be the origin of the development of a pathological atopobiome in different tissues including the brain (Kelly et al., 2015), the spine and discs (Teichtahl et al., 2015) and the shoulder (Hajishengallis, 2015). The way the bacteria reach the infiltrated organs is still in debate although data suggest that they migrate in an ‘invisible state of dormancy’ (Malka et al., 2016). The dormant state of the bacteria prevents immune detection and facilitates migration and infiltration in organs with a low pO2 level such as the intervertebral disc and the shoulder capsule, and even more in sedentary people (de la Serna et al., 2021). Therefore, a combination of low-grade inflammation and the possible presence of a subclinical infection could explain the observed inflammation-fibrotic cascade in FS.

The inflammatory phase can be considered a protective event but it can become a threat when it is exacerbated (Potgieter et al., 2015); for this reason, inflammation has been linked to several degenerative diseases as well as mitochondrial alterations, with mitochondria playing an important role in pro-inflammatory signaling (Campos et al., 2003). Strategies aimed at controlling excessive oxidative stress in mitochondria may represent preventive and therapeutic interventions in inflammation (López-Armada et al., 2013). Impaired mitochondrial function is associated with several acute and chronic inflammatory diseases (López-Armada et al., 2013). A recent review has shown how mitochondrial dysfunction can end up appearing in many different pathologies, such as fibromyalgia, chronic fatigue syndrome, diabetes, some types of cancer, and neurodegenerative diseases (e.g., Parkinson’s or Alzheimer’s). In patients with FS, it seems logical to suspect mitochondrial dysfunction as a consequence of the observed alterations in glucose metabolism, insulin resistance, lack of shoulder mobility, a low oxygen level and the level of free radicals as all known risk factors for mitochondrial dysfunction (Casanova et al., 2023), as well as in chronic shoulder pain (Hamed-Hamed et al., 2023).

If LGI and subclinical infection contribute to FS, interventions targeting both entities should prevail in patients suffering from FS. Although common opinion is that the use of NSAIDs as anti-inflammatory intervention could help to resolve FS, data are confounding (Ewald, 2011). NSAIDs have been part of medicine since decades and their role is based on the assumption that the metabolites produced out of arachidonic acid (AA) (as part of the debris in tissue injuries) are deleterious for wound healing and cause inflammation (Wang et al., 2021). NSAIDs inhibit COX-2 enzymes responsible for the production of different metabolites of AA, including prostaglandin E2 (PgE2, 82). PgE2 has long been considered a pro-inflammatory eicosanoid (Kawahara et al., 2015). Studies from Hangai (Hangai et al., 2016) and more recently Avramia (Avramia et al., 2021) show that PgE2 should be considered an immune-suppressive damage associated molecular pattern (DAMP) and is probably therefore the most abundant eicosanoid in an acute inflammatory environment (Hangai et al., 2016). The notion that inflammation is some ‘bad’ invention of evolution has been the driver of the search for anti-inflammatory drugs for decades. Serhan (Serhan, 2017) warns for the possible immune-suppressive activities of anti-inflammatory drugs and its iatrogenic effect on the development of chronic inflammation by preventing resolution (Serhan, 2017). Parisien et al. (Parisien et al., 2022) showed the possible negative impact of NSAIDs use after acute injury on resolution and possibly causing chronic pain syndromes (Parisien et al., 2022). The study performed a transcriptome-wide analysis in peripheral immune cells of 98 individuals suffering from acute low back pain, and followed up for 3 months (Parisien et al., 2022). The main finding was that neutrophil activity in inflammation was protective against the development of chronic pain and thousands of genetic transcriptional changes were observed only in those participants with resolved pain and none in those with persistent pain (Parisien et al., 2022). Further research elicited that mice receiving NSAIDs showed the same absence of transcriptional activity as the immune cells of the participants with persistent pain. Retrospective analysis of individuals reporting acute low back pain in the UK Biobank identified an increased risk of the development of chronic pain in patients using NSAIDs (Parisien et al., 2022).

To our knowledge, there is no research done on the possible negative effects of the use of NSAIDs in persons suffering from FS. Despite pain relief at start, the use of NSAIDs and its anti-inflammatory effects could be counteractive for long outcome of FS. Already in 2011 (Ewald, 2011) evidence of the use of NSAIDs in people suffering from FS was limited and hard evidence still lacks.

We propose to use a much more integrative approach in the treatment of patients suffering from FS. In line with other disciplines, it is perhaps time to recover the knowledge about the way the immune system normally solves injury and infection successful (Truchetet and Pradeu, 2018).

As knowledge advances about the way fatty acids influence immune activity, new lipid formulas with beneficial effects have been developed. Those lipids are nutritionally adapted and are useful for improving inflammation-based pathologies promoting a faster recovery and reducing the use of anti-inflammatory drugs that can produce serious adverse effects (Serhan, 2017). This benefit has led fatty acids, especially n-3 PUFAs and oleic, to be considered as immuno-nutrients (Mesa et al., 2006). Likewise, olive oil phenolics have been shown to reduce the generation of reactive oxygen species (ROS) in phagocytic cells, *in vitro*, and inhibit the activity of the enzyme 5-lipoxygenase involved in pro-inflammatory events (Shibutami and Takebayashi, 2021). This approach may allow for the identification of dietary biomarkers and a deeper understanding of metabolic dynamics and the resulting impact on health (Vazquez-Aguilar et al., 2023). The use of omics-science could help to find the exact immunological players in people suffering from FS. The search for the presence of super autoantigens and their antibodies is needed to support our new hypothesis presented earlier in this paper.

## 4 Metabolomics and proteomics in pain and chronic conditions as proxy for FS

The science of metabolomics studies low molecular weight chemical compounds that exist in biological tissues or cells because of genetic changes or physiological or pathological metabolic conditions (Cui et al., 2017).

Proteomics is defined as the complete evaluation of the structure and function of proteins to understand the nature of an organisms. The main purpose of proteomics is to find essential biomarkers that support mechanistic pathways in health and disease (Al-Amrani et al., 2021). Proteomics provides a better understanding of the structure and the function of an organism than genomics. Nevertheless, it is much more complicated than genomics because of the immense number of estimated proteins in humans (>million) opposite to the relative small number of genes (22.000). Proteomics could be used to verify our hypothesis about why DM1 is more associated with FS than DM2. That research should find proteins of an immune reaction related with T lymphocytes and the presence of GAD65 or other super autoantigens and their antibodies. To our knowledge such research is lacking in the international scientific literature and so for now it is still speculative.

One manuscript describes the proteomics of FS in different parts of the shoulder capsule and in different types of patients (Kawahara et al., 2015). Protein spectrum was measured in different compartments of the shoulder capsule in twelve patients suffering from primary FS with severe stiffness compared with seven patients presenting FS with a rotator cuff tear as the control group (Kawahara et al., 2015). There were important differences found between the two groups of patients and several proteins were highlighted as clinical important (Kawahara et al., 2015). Firstly, there are significant differences in presence of proteins related with P13K-Akt and PPAR signaling which are essential compounds in insulin and leptin metabolism (Giorgino et al., 2009; Thon et al., 2016). Patients with primary FS present increased P13K-Akt and PPAR signaling compared with the rotator cuff group (Hagiwara et al., 2018). Increased activity of both signaling pathways suggest greater presence/activity of insulin and leptin as part of the pathophysiology of primary FS and related with insulin and leptin resistance (Thon et al., 2016; Huang et al., 2018). Persons with rotator cuff damage present a proteome much more related with direct damage and less with metabolic disturbances. The latter conclusion is based on the presence of proteins that signal *staphylococcus aureus* infection, antigen processing/presentation, and lysosome and phagosome activity (Kawahara et al., 2015). Phagosome and lysosome activity elicit the presence of damage associated molecular patterns and pathogen associated molecular patterns (Roh and Sohn, 2018; Khorshidian et al., 2022). Damage and possible pathogen infiltration by opening the blood/capsule barrier seem to be the main causes of the symptomatology of FS after rotator cuff damage. This could mean that patients suffering from primary FS need treatment much more focused on lifestyle changes and specific metabolic interventions to solve insulin and leptin resistance, whereas people with traumatic FS demand a much more orthopedic treatment. The writers of this study also conclude that primary and secondary FS show different etiology and pathophysiology, meaning that interventions should also differ (Kawahara et al., 2015).

It is obvious that only one proteomics study in people suffering from FS cannot be used as golden standard. Much more studies are necessary to determine and identify the exact mechanisms and etiology of primary and secondary FS, but the first step has been made.

Not published as a proteomics research paper but as a pathology manuscript, Hand et al. (Hand et al., 2007) showed the presence of multiple immune cells in the shoulder of 22 conservative therapy resistant patients suffering from primary FS. Mast cells presence was highest followed by T lymphocytes, B lymphocytes and macrophages, evidencing a state of chronic inflammation in primary FS (Hand et al., 2007). Mast cells regulate fibroblast proliferation, an interaction that suggests that the inflammatory state leads to fibrosis through intermediation of mast cells (Hand et al., 2007). The presence of T and B lymphocytes as part of the acquired immune system gives rise to the existence of an autoimmune reaction in the frozen shoulder against the fibrotic tissue (Hand et al., 2007), although other hypotheses could be emphasized. More research will shed light on our hypothesis in which anxiety, fear and depression are related with increased risk for the activation of T lymphocytes against GAD65, diabetes type 1, Hashimoto and perhaps FS (Nataf, 2017).

Proteomics science in FS is still very limited, so metabolomics can add only limited knowledge to the etiology and pathophysiology of FS. Perhaps the best way to identify biomarkers for FS is the use of metabolomics studies related with global chronic pain conditions and translate those studies in comparative pathology to FS (Aroke and Powell-Roach, 2020). Mantyselka et al. observed that in a group of individuals with chronic musculoskeletal pain the circulating levels of ornithine and amino acids were increased when compared to another group where individuals reported no pain or their pain was not persistent (Mäntyselkä et al., 2017). These results are consistent with a 2019 study investigating biomarkers and glutamate changes in fibromyalgia patients suffering from chronic musculoskeletal pain. The metabolites found to be altered included ornithine, L-arginine, Nε-methyl-L-lysine, L-glutamate, L-glutamine and asymmetric dimethylarginine (ADMA) (Clos-Garcia et al., 2019). Moreover, metabotropic glutamate receptors could also play a role in neuropathic pain (Osikowicz et al., 2013). In the study by Finco et al. higher levels of choline and phosphocholine, citrate, alanine and taurine were seen in patients with neuropathic pain when compared to patients with nociceptive pain (Finco et al., 2016).

Further studies show that lipid metabolism dysregulation is involved in the persistence of pain (Ma et al., 2022). The study by González et al. suggests that serum lipid markers, specifically sphingomyelins and triacylglycerols, are associated with chronic pain (Gonzalez et al., 2022).

These findings could eventually be used to develop primary preventive interventions (Teckchandani et al., 2021).

The evidence that idiopathic FS is associated with glucose and lipid metabolic diseases is increasing. One study observed that some proteins, such as adipokines, adiponectin, leptin and resistin, which are involved in metabolism, have been linked to the development of frozen shoulder (Mäntyselkä et al., 2017).

Moreover, Bunker T. et al. focused on hyperlipidemia as a possible risk factor for frozen shoulder after observing clinical relationships between it and Dupuytren’s contracture (Al-Amrani et al., 2021); this aforementioned study supports the hypothesis that elevated serum lipid levels are associated with frozen shoulder, and demonstrates that low-density hypercholesteremia and lipoprotein hypercholesteremia are progressively associated with frozen shoulder (Al-Amrani et al., 2021).

Again another study found higher expression levels of TNC, which is a lipoprotein involved in cell adhesion, fibroblast migration and other processes related to tissue remodeling and wound healing, in synovial capsule samples from patients with adhesive capsulitis (Mertens et al., 2022).

Molecular biology studies have shown frozen shoulder capsular changes indicating that angiogenesis, inflammatory cell infiltration and expression levels of inflammatory cytokines, such as interleukin (IL1-IL6) increase in frozen shoulder (Hagiwara et al., 2018).

To the best of our knowledge, preliminary results have only been reported in one study regarding this and it showed that the serum total cholesterol level may be related to shoulder stiffness (de La Puerta Vázquez et al., 2004; Sung et al., 2014; Lee et al., 2019).

Etiological and mechanistic research currently lack new hypothesis explaining the spontaneous occurrence of primary FS whereas traumatic secondary FS is much less mystical. Our new perspective of the development of FS, based on the interaction between the brain and the immune holds promise when confirmed by further investigations (Nataf, 2017; Dantzer, 2018).

## 5 Proposed interventions for frozen shoulder

Current therapies for the treatment of FS are both surgical and non-surgical, although no definitive management model is available yet (Challoumas et al., 2020).

Treatment choice depends mostly on expert opinions and is mostly based on qualitative experience instead of quantitative and recent research (Bridgman, 1972). There is no consensus about the best common treatment for patients with FS (Johnson et al., 2007) and therefore new perspectives are urgently needed.

We propose to use a drug-free treatment protocol based on the different mechanisms and etiological factors known to be part of FS. As chronic anti-inflammatory pharmacological interventions can be deleterious (Johnson et al., 2007; Challoumas et al., 2020), the use of nutrition as medicine could provide valid effects on the inflammatory state or even on the mental state of patients suffering from FS (Brain et al., 2019). Furthermore, experimental and epidemiological data indicate that changes in the source of lipids consumed in the diet can modify the fatty acid composition of many cell types, including cells involved in the development of many inflammatory and immunological diseases (Shibutami and Takebayashi, 2021; Hamed-Hamed et al., 2023). Hence, the use of dietary strategies as part of the FS treatment could be indicated.

The use of pre- and/or certain probiotics, as remedy against disturbances of the microbiome, could provide both acute and chronic inflammatory relief (Ho et al., 2014) through regulation of the immune response and increase in the production of IL-10 (Ho et al., 2014). IL10 is capable of inhibition of cytokine and inflammatory chemokine production (Akdis and Akdis, 2014). *Lactobacillus* plantarum (L. plantarum), a common used probiotic bacterial strain, inhibits the production of pro-inflammatory cytokines such as TNF- α and IL-6 (Ahn et al., 2019). The possibility of infiltration of pathogenic virus or bacteria as part of the pathophysiology of FS makes it legitimate to propose the use of lactoferrin as a natural antimicrobial, anticarcinogenic, antioxidant, and anti-inflammatory substance (Zimecki and Kruzel, 2007).

Other less known interventions could serve multiple purposes of which the use of therapeutic hypercapnia is perhaps the most indicated one (Tolstun et al., 2022). The use of therapeutic hypercapnia, the search for short term increase of CO2 in the bloodstream up to 50 mmHG by breathing exercises or CO2 devices with a partial pressure of 60–70 mmHG, has shown surprising effects on diet related behavior, blood circulation in the brain, integrity of the blood-brain barrier and immune activity in mice and human (Yang et al., 2019; Tolstun et al., 2022; Tzeng et al., 2022).

The life of the naked mole rat, known for its longevity and resistance against multiple diseases, was used as a model for life expectancy enhancing in rodents (Tolstun et al., 2022). The naked mole rat lives in a natural intermittent hypoxia/hypercapnia environment. Imitating this environment, other rodents also slowed down their metabolism, presented accelerated skin wound healing and reduced their calorie intake with 30%–40% spontaneously (Tolstun et al., 2022). If these results would be confirmed in human we would have a simple way to reduce over-eating as one of the most important deleterious lifestyle factors in modern life. Therapeutic hypercapnia further has profound effects on the immune system. The study of Tzeng (Tzeng et al., 2022), shows that the use of therapeutic hypercapnia ameliorates acute cellular rejection in the skin of a mouse acute allograft rejection model by suppressing the expression of proinflammatory cytokines and neutrophil infiltration, inhibiting T cell activation and accumulation, and inducing Treg cell differentiation (Tzeng et al., 2022). If these effects are confirmed in humans, then the use of a plastic bag rebreathing strategy could be effective to (partially) counter chronic inflammation and reduce calorie intake spontaneously. Already in 1995 (Yang et al., 2019) it was shown that 1–2 min rebreathing in a plastic bag could by slightly increasing CO2 levels in the blood, improve retinal arterial obstructive diseases in young patients (Harino et al., 1995).

Therapeutic and permissive hypercapnia are often used in the same context although there are important differences between them. Therapeutic hypercapnia is based on active inspiration of air with higher pCO2 than normal, whereas permissive hypercapnia is the tolerated CO2 level when lungs are less stressed (Lee et al., 2019). We propose ‘plastic bag rebreathing technics’ as a simple and effective way to increase pCO2 in blood and produce the beneficial effects of hypercapnia. The possibility that this technic causes any damage when controlled for time and tolerance is minimum. Nevertheless, hypercapnia can be damaging as evidenced during the COVID-19 pandemic. During the pandemic, health professionals came in situations in which they used a plastic bag over their head to protect themselves against possible infection with SARS-CoV-2 (Bakirtzi et al., 2016). This way rebreathing their own breath was forced. Three volunteers were used as a model for tolerance measurements and negative effects of forced ‘therapeutic’ hypercapnia (Lee et al., 2020). CO2 levels in the bag increased almost immediately and resulted in significant hypercapnia at termination. Tolerance was reached at 5 min and was produced by breathlessness, anxiety and feelings of distress (Lee et al., 2020). We propose intermittent therapeutic hypercapnia exercises with a length of 90 s and several times per day.

The proposed interventions are drug free, under control of the patients and promising when combined with knowledge transfer about pain, fear, anxiety and depression as assessed by multiple studies of the group of Lorimer Mosely and David Butler (Moseley and Butler, 2015).

## 6 Conclusion

This study features an integrative view based on the current scientific evidence on FS pathology. Frozen shoulder has been observed to share mechanisms, such as low-grade inflammation and multiple metabolic disturbances, with pathologies such as diabetes and Hasimoto syndrome. Etiology of FS with autoimmune diseases also share essential topics such as the simultaneous presence of immune challenges and psycho-emotional distress at the start of the disease. Knowledge about the interplay between the immune system and the brain gives rise to the development of new treatments for people suffering from FS. Identification of the mechanisms that explain the association between an increased risk for the development of FS in people suffering from type 1 diabetes, Dupuytren, Hashimoto, and axial Spondylarthritis are missing, although several hypotheses have been elaborated in this review. Disorders of the GABA-ergic nervous could be (co-) responsible for the possible association between the mentioned maladies and FS risk. The COVID-19 pandemic increased the incidence of the development of FS, type 1 diabetes, Hashimoto and Dupuytren significantly (de Mello et al., 2023). Fear, anxiety and depression seem were associated with the increased incidence of the mentioned maladies and all three states are related changes in the GABA-ergic nervous system (Kupcova et al., 2023). A possible neurogenic background of FS has recently been elucidated in a small study with 10 patients who received a central nervous system combined treatment with significant improvement (Mena-Del Horno et al., 2022). We further propose the possibility that FS, just as the associated maladies has an autoimmune background. The striking increase of FS when suffering from several autoimmune diseases, including but less mentioned axSA, suggests a possible auto-immune background for FS. Both explanations do not exclude one of them. The opposite is true. The way the brain and the immune system interplay when faced with simultaneous immune and psycho-emotional challenges increased the possibility that immune cells are primed against super-autoantigens in the brain and it are these antigens that are also present in peripheral tissues and organs. If those primed T lymphocytes can destroy Beta-cells of Langerhans, it is plausible that these Teffector cells can destroy other tissues when faced with the same antigen. There are important indications for the presence of a possible neuroimmune connection as cause for FS and several other diseases; nevertheless this new perspective about the etiology of FS has to be confirmed with new mechanistic research.
